# Evaluation of Brix Refractometry to Estimate Immunoglobulin G Content in Buffalo Colostrum and Neonatal Calf Serum

**DOI:** 10.3390/ani11092616

**Published:** 2021-09-06

**Authors:** Melania Giammarco, Matteo Chincarini, Isa Fusaro, Anna Chiara Manetta, Alberto Contri, Alessia Gloria, Lydia Lanzoni, Ludovica Maria Eugenia Mammi, Nicola Ferri, Giorgio Vignola

**Affiliations:** 1 Faculty of Veterinary Medicine, University of Teramo, Loc. Piano d’Accio, 64100 Teramo, Italy; mgiammarco@unite.it (M.G.); mchincarini@unite.it (M.C.); acmanetta@unite.it (A.C.M.); agloria@unite.it (A.G.); llanzoni@unite.it (L.L.); gvignola@unite.it (G.V.); 2 Faculty of Biosciences and Technologies for Agriculture Food and Environment, Via Balzarini 1, 64100 Teramo, Italy; acontri@unite.it; 3 Department of Veterinary Medical Sciences, University of Bologna, Via Tolara di Sopra 50, Ozzano Emilia, 40064 Bologna, Italy; ludovica.mammi@unibo.it; 4 Istituto Zooprofilattico Sperimentale dell’Abruzzo e del Molise ‘G. Caporale’, Campo Boario, 64100 Teramo, Italy; n.ferri@izs.it

**Keywords:** failure of passive immunity transfer, newborn, buffalo, digital brix refractometer, ELISA

## Abstract

**Simple Summary:**

The protective effects of colostrum in relation to the incidence and severity of newborn ruminant diseases are well established. Neonatal calf depends on the timely supply of high-quality colostrum to prevent the failure of passive transfer of immunoglobulins (Ig), which has been linked to increased risk of different diseases and mortality in early stages of life. Despite the relevance of Buffaloes (*Bubalus Bubalis*) in world dairy production, the available knowledge regarding colostrum quality management remains scarce for this species. Therefore, the objective of this study was to evaluate the utility of a simple and rapid tool such as a digital Brix refractometer to estimate colostrum quality and for predicting the success of passive transfer of immunoglobulin G (IgG) in Buffalo calves. For this aim, correlation analysis was performed between Brix results and ELISA-IgG determination from colostrum and serum samples. A strong correlation was found between Brix measurements and IgG content in colostrum and serum samples. Moreover, cut point values for Brix measurements for colostrum and serum samples were determined. Brix refractometry was found to be an acceptable tool for on-farm estimations of colostrum quality and passive immunity transfer (PIT) in Buffalo calves.

**Abstract:**

Brix refractometry has been widely demonstrated to be a useful tool for monitoring colostrum management program and passive immunity transfer (PIT) in Bovines, but its suitability has never been verified in Buffalo. Therefore, the objective of this study was to evaluate the utility of a simple and rapid tool such as a digital Brix refractometer to estimate colostrum quality and for evaluating the success of passive transfer of immunoglobulin G (IgG) in Buffalo calves. The optimal cut points levels for Brix Refractometry for distinguishing good- and poor-quality colostrum and for assessing the adequacy of passive immunity transfer in calves were determined. For this aim, 26 first-milking maternal colostrum (MC) were collected from first-calf heifers. Blood samples were obtained from their calves at birth (T0) and 72 hours after (T3). Colostrum and Serum IgG content were determined by indirect enzyme-linked immunosorbent assay (ELISA), whereas total protein (TP, g/dL) and percentage Brix (%Brix) by means of a digital Brix refractometer. The mean colostrum IgG was 64.9 ± 29.3 mg/mL. The mean serum %Brix at T3 was 9.6 ± 0.9 %. The mean serum IgG content at T3 was 11.1 ± 2.0 mg/mL. Pearson’s correlation coefficient (*r_p_*) was determined between Brix and ELISA measurements: colostrum %Brix showed a significant correlation with serum %Brix (*r_p_* = 0.82, *p* < 0.001); serum %Brix was highly correlated with serum TP (STP, g/dL) (*r_p_* = 0.98, *p* < 0.001) and serum IgG (mg/mL) (*r_p_* = 0.85, *p* < 0.001). A cut point of 18% Brix to estimate samples of MC ≥ 50 mg/mL from first-calf heifers was more appropriate for the buffalo. A cut point of 8.4% Brix resulted in the greatest percentage of calf serum samples being correctly classified. Based on our findings, a digital Brix refractometer could be a useful tool to monitor colostrum quality and to estimate PIT in Buffalo calves.

## 1. Introduction

Newborn ruminants depend on maternal colostrum to provide them with nutrients and bioactive compounds needed to sustain life [1]. Colostrum represents a critical source of immune-protection for newborn calves [2]. As ruminants, buffalo calves are born agammaglobulemic and highly dependent on efficient enteral uptake of maternal immunoglobulin G (IgG) from colostrum to protect them from infections until their own immune system has developed [3,4,5].

The period during which a calf can acquire immunoglobulin through intestinal absorption is limited after birth: Their ability to absorb Ig decreases rapidly after 12 h with mean gut closure time of 24 h after birth [6,7]. Therefore, the ingestion of good-quality colostrum within the first 24 h of life is essential for the future health and performance of calves [8]. Insufficient ingestion or absorption of colostral IgG results in failure of passive immunity transfer (FPIT) [3,9], which occurs when serum IgG concentration is less than 10 mg/mL [10] or serum protein levels are less than 5.2 to 5.5 g/dL. Prevention of FPIT is achieved by timely feeding of adequate quantities of colostrum that contains a minimum of 50 mg of IgG/mL as measured by radial immunodiffusion. Because newborn calves have a relatively short period before gut closure, there is a clear need for a rapid on-farm test to assess immune status [11]. Calves with FPIT are in fact more susceptible to infectious diseases and have higher morbidity and mortality rates, as well as diarrhea and respiratory disease [12]. The FPIT prevalence lesser than 10% is a reasonable goal in ruminants [13]. Neonatal diarrhoea is one of the main causes of calf death with higher mortality rates in the first weeks of life, thus affecting the welfare and production efficiency of buffalo farms. In most buffalo farms, the incidence of calf loss from birth to weaning is usually between 10 and 20% and this percentage can increase to more than double this value if animals are reared under sub-optimal conditions [14]. The controlling of calf mortality is one of the most important factors for increasing profits from dairy farming. An efficient calf management is the first step to guarantee an adequate productive improvement of the herd and provide the foundation for a profitable dairy enterprise [15]. Thus, in a calf management program, the assessment of the concentration of IgG in colostrum and calf serum is crucial in order to monitor the efficacy of passive immunity transfer. Different laboratory techniques have been applied to estimate colostral immunoglobulin transfer to calves [16,17,18,19]. Weaver et al. (2000) [10] reported single radial immunodiffusion (sRID) [20,21,22] and enzyme-linked immunosorbent assay (ELISA) to be the only true and direct measures of colostrum or serum IgG [23,24,25]. IgG can also be measured indirectly by rapid on farm methods including refractometry, which represents an inexpensive, rapid, and accurate test that has shown a close relationship with IgG measured by laboratory methods for colostrum and serum samples in Bovines [1,2,26,27,28,29], goat and sheep [29]. Brix refractometers have been promoted by the dairy industry as an effective on-farm rapid tool to estimate colostral IgG and colostrum quality [30]. More recently, it has also been proposed to assess IgG in calves’ saliva, as a non-invasive method to detect FPIT (Johnsen et al., 2019) [31].

Even though a timely supplying of high-quality colostrum is crucial also for buffalo calves, no studies are available regarding the use of a Brix refractometer for estimating colostrum quality or PIT in this species. 

Therefore, the aim of the present study was to evaluate the suitability of a digital refractometer for a rapid estimation of colostrum quality and for the evaluation of passive transfer of immunity from Buffalo dams to calves. For this objective, Brix refractometry readings were compared to ELISA quantitative IgG determinations in colostrum and serum samples. Moreover, diagnostic test characteristics for using the Brix refractometer with different test break points for colostrum and serum samples were calculated.

## 2. Materials and Methods

### 2.1. Animals and Housing

The study was performed at the experimental farm of the “Istituto Zooprofilattico Sperimentale dell’Abruzzo e del Molise” (Italy), from December 2018 to March 2019. All the procedures were approved by the Italian Health Ministry (nr. 498/2016-PR of the May 20, 2016, prot. 781AA.3.EXT.2 of the August 11, 2016).

To reduce the variability of the subjects considered in the trial, only pregnant heifers of Italian Mediterranean Buffalo (*Bubalus bubalis, subspecies bubalis*), housed in similar conditions, and with the same food regimen, were included in this study. Furthermore, buffalo cows in which obstetric procedures were performed (assisted calving) were excluded by the trial.

The heifers were kept in group housing and moved into individual calving pens from 15 days before to 10 days after parturition. The individual pens (3.5 × 3.5 m) allowed social and visual contact and were equipped with straw bedding. 

The heifers were fed with commercial concentrate and hay and they had ad libitum access to an automatic drinker. Estrus was synchronized as previously reported [32] and the buffaloes were artificially inseminated at the age of 19–21 months. The calving day was predicted on the basis of ovulation day, and the onset of calving was monitored using an intravaginal devices as described [33]. This latter allowed us to have a precise assessment of the calving time, providing initial care to the newborn calves (umbilical cords were treated with 2% iodine solution) and assessing clinical parameters [34]. The calves were allowed to nurse the dam freely for the entire experimental period. 

Animals underwent thorough daily physical examination and were considered clinically healthy when they did not present any alterations on physical examination.

### 2.2. Samples Collection

First milking colostrum samples were collected from all mammary quarters of individual Buffalo cows immediately after parturition (within 30 minutes) in 50 mL polypropylene tubes, immediately frozen at −20°C, and transferred in this state to the Faculty of Veterinary Medicine of Teramo (Italy) for later analysis. 

Whole blood samples were collected from calves (*n* = 26) before the first colostrum feeding (T0) and at 72 hours (T3) after birth by jugular venipuncture using a 20-gauge, 1-inch hypodermic needle into sterile, plastic, Vacutainer tubes without anticoagulant (BD Vacutainer, Becton Dickinson and Co.). Blood samples were all collected by the same experienced veterinarian. Samples were immediately centrifuged (Refrigerated Megafuge, 1.0 R, Heraeus, Kendro, Langenselbold, Germany) at 1500× *g* for 15 min in order to collect the serum for assessing Brix percentage (%), total protein (TP) (g/dL) and IgG (mg/mL) content. The serum samples were aliquoted and then stored at −20 °C until analyses. The specific time point after calf birth (T3) was chosen to represent optimum serum IgG concentrations [35].

### 2.3. Brix Measurements of Colostrum and Serum Samples

The digital Brix determined the Brix score of the colostrum or serum by shining a light through the sample in the well, measuring the index of refraction, and presenting the reading in Brix units on a digital scale, as described by Bielmann et al. (2010) [2]. The digital Brix refractometer is generally used to measure the percentage of sucrose in liquids, and when used in non-sucrose-containing liquids approximates the amount of total solids (TS, %) [27]. In the present study, thawed colostrum samples were vortexed for 10 s and then analyses of %Brix and TP was performed using a commercial digital refractometry instrument (Palme Abbe, Misco Digital Dairy^TM^ PA203-DD3, Digital Refractometer, Solon, OH, USA) with a range of 0 to 85% Brix and an automatic temperature compensation mechanism to ensure accurate measurements without recalibration at different ambient working temperatures. The accuracy of the instrument was ± 0.1% Brix at 20 °C. Before use, the refractometer was calibrated to zero using distilled water. In our study, a 0.3 mL of colostrum and serum sample was used for the measurement of Brix percentages and TP. According to the literature, MC with a value between 18 and 23% Brix is an appropriate cut-point for good quality colostrum [2,26,27,36]. Calf serum with a value between 7.8 and 8.4% Brix has been proposed as indicative of the success of PIT [37,38].

### 2.4. Colostrum and Serum Immunoglobulin G Quantitative Analyses 

The ELISA method used to quantify colostrum and calf serum IgG concentration was performed according to manufacturer’s protocol (IgG ELISA Core Kit, Koma Biotech INC., Seoul, Korea) and modifications as described by Singh et al. (2011) [39]. According to the literature, serum samples with IgG content < 10 mg/mL were considered indicative of FPIT [3,10]. As previously stated, colostrum samples with IgG content < 50 mg/mL were considered of poor quality [26].

### 2.5. Statistical Analysis

Statistical analysis was performed with SPSS 26.0 software (IBM Company Headquarters, Chicago, IL, USA). A G-power analysis was performed in order to calculate the minimum number of animals that should be included. G-power analysis showed that the minimum number of animals needed was 26, considering an effect size (Cohen coefficient) of 0.84, an alpha value of 0.05, and a power of 0.89.

Normality and homogeneity of data were examined graphically and checked with the Kolmogrov–Smirnov test. Data are presented as mean values ± standard deviation (SD). Colostrum and serum Brix refractometer measurements expressed in %Brix units or g/dL were plotted against ELISA results (mg/mL). From these distribution plots, Pearson’s correlation coefficients (*r*-values) were determined. Correlation coefficients obtained were considered high only when (*r*) ≥0.50. *P*-value was considered statistically significant at ≤0.05. Paired samples *t*-Test was performed to compare mean serum Brix (%), STP (g/dL), and ELISA-IgG measured at T0 and T3. 

Test characteristics (sensitivity and specificity) of the brix refractometer were calculated by means of Excel 2010 (Microsoft Corp., Redmond, WA). The sensitivity (Se), specificity (Sp), accuracy, positive predictive values (PPV) and negative predictive values (NPV) (95% CI), false negative, and false positive were determined at different cut points. 

Prior to calculation of Se and Sp, the ELISA and Brix% measurements from the colostrum were compared in 2 × 2 table at %Brix cut points of 18, 19, 20 and 23%. 

Sensitivity was defined as the proportion of poor-quality samples (<50 mg/mL) that were correctly identified as such, and Sp was defined as the proportion of good-quality samples (≥50 mg/mL) that were correctly identified as such. Accuracy was defined as the proportion of colostrum samples that were correctly classified. The PPV was the proportion of test-positive samples that truly had IgG < 50 mg/mL and the NPV was the proportion of test-negative samples that truly had IgG ≥ 50 mg/mL.

Sensitivity and specificity were summed, and the highest combined value was used to determine the appropriate cut-points for the Brix refractometer [40] in order to minimize the risk of both false negatives and false positives [41]. 

The same approach was applied for the serum sample at %Brix cut points of 7.8, 8.0, and 8.4%. Sensitivity was defined as the probability of a test result indicative of FPIT for a serum sample with IgG < 10 mg/mL. Specificity was defined as the probability of a test result indicative of adequate passive transfer for a sample with IgG ≥ 10 mg/mL. 

The best cut point value was defined as the one that gave the optimal combination of Se, Sp, and accuracy.

## 3. Results

### 3.1. Descriptive Statistics: Brix and ELISA Measurements 

Based on the criteria of inclusion, in this study 26 dams (623 ± 19.2 kg of body weight; 3.2 ± 0.3 years old) and their calves (12 females and 14 males) were enrolled in the experiment. Descriptive statistics of colostrum and serum samples measurements are shown respectively in Table 1 and Table 2.

None of the included buffalo calves showed signs of disease or discomfort during the time of the study. All data were normally distributed. Mean time between birth and calves first suckle was 212 ± 110 min, as reported by Lanzoni et al. (2020) [42]. The mean IgG content measured by ELISA in first-milking maternal colostrum was 64.9 ± 29.3 mg/mL with a range from 21.0 to 110.0 mg/mL. According to the ELISA assay, 77% of colostrum samples had IgG concentrations above the cut point of 50 mg/mL (good quality). A significant increase of serum Brix, serum total protein, and IgG were found between T0 and T3 (*p* < 0.001), as summarized in Table 3. Of the serum samples, 85% had IgG concentrations ≥ 10 mg/mL, consistent with adequate passive transfer. 

### 3.2. Correlations

Correlation analysis results and *p*-value between the digital Brix refractometry measurements and ELISA determinations on colostrum and serum samples are reported in Table 4.

**Figure 1:**
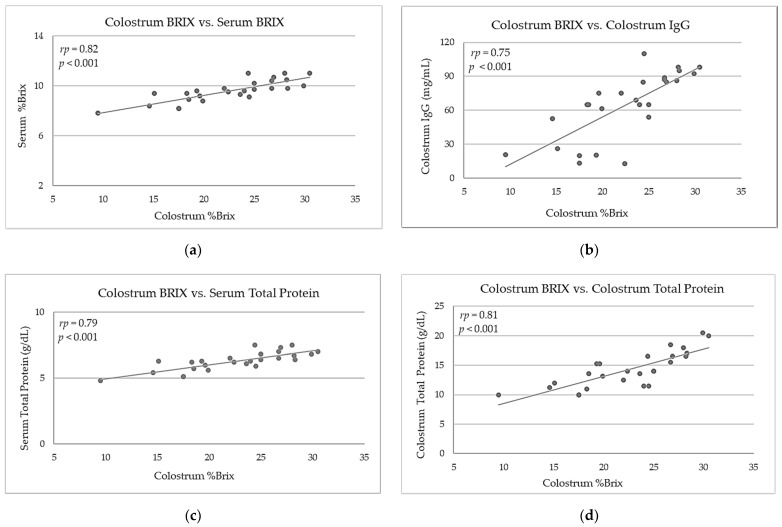
(**a**) Relationship between colostrum %Brix and calf serum %Brix (obtained 72 hours after birth) (*r_p_* = 0.82, *p* < 0.001); (**b**) Correlation between colostrum %Brix and colostrum IgG (mg/mL) (*r_p_* = 0.75, *p* < 0.001); (**c**) Correlation between colostrum %Brix and serum total protein (g/dL) (*r_p_* = 0.79, *p* < 0.001); (**d**) Relationship between colostrum %Brix and colostrum total protein (g/dL) (*r_p_* = 0.81, *p* < 0.001). *r_p_* is the Pearson’s coefficient of correlation.

**Figure 2:**
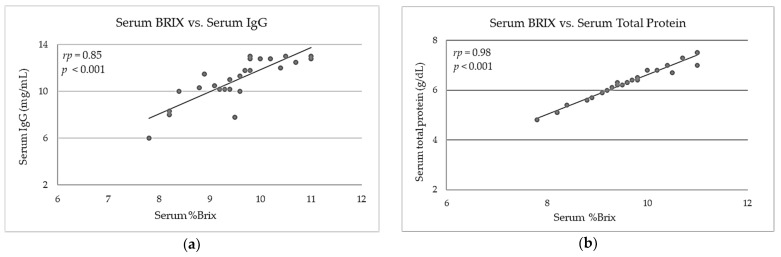

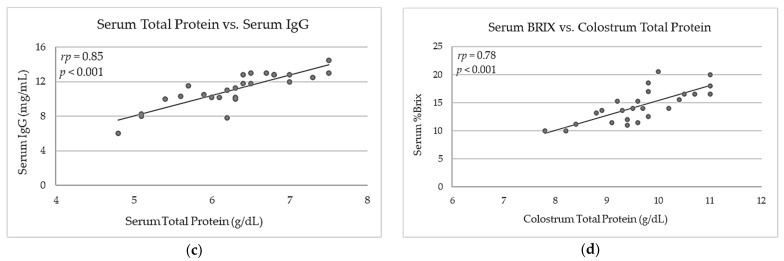
(**a**) Relationship between calf serum %Brix (at 72 h) and serum IgG (mg/mL) (at 72 h) (*r_p_* = 0.85, *p* < 0.001); (**b**) Correlation between calf serum %Brix and serum total protein (STP, g/dL) (*r_p_* = 0.98, *p* < 0.001); (**c**) Correlation between serum total protein (g/dL) and serum IgG (mg/mL) (*r_p_* = 0.85, *p* < 0.001); (**d**) Relationship between serum %Brix and colostrum total protein (g/dL) (*r_p_* = 0.78, *p* < 0.001). *r_p_* is the Pearson’s coefficient of correlation.

The distributions of colostrum %Brix vs. serum %Brix, colostrum %Brix vs. colostrum and serum IgG (mg/mL), and colostrum %Brix vs. colostrum total protein (g/dL) are plotted in Figure 1 (a), (b), (c), and (d) respectively. The distributions of serum %Brix versus STP (g/dL), serum %Brix vs. serum total protein (g/dL), serum total protein (g/dL) vs. serum IgG (mg/mL), and colostrum IgG (mg/mL) vs. serum IgG (mg/mL) are shown in Figure 2 (a), (b), (c), (d), respectively. 

### 3.3. Diagnostic Test Characteristics for Colostrum and Serum Samples 

Diagnostic test characteristics were assessed for the digital Brix refractometer at different test break points for colostrum and serum samples (Table 5 and Table 6). 

Four different cut point levels for colostrum Brix were evaluated: 18, 19, 20, and 23%. The calculated sensitivity and specificity of the instrument were used to identify appropriate cut points to be proposed for colostrum from buffalo heifers. The highest combined sensitivity and specificity for the detection of adequate quality colostrum, as determined by ELISA, occurred at 18% Brix (Table 5).

Moreover, test characteristics of sensitivity and specificity of Brix refractometer were determined for the assessment of FPIT (serum IgG < 10 mg/mL) at different cut points, namely 7.8%, 8%, and 8.4% (Table 6).

## 4. Discussion

To the authors’ knowledge, this is the first experimental study applying the Brix refractometer to detect good quality colostrum and the success of PIT in Buffalo calves. For this reason, the authors referred mainly to studies focusing on the use of refractometry in bovines or small ruminants for discussing their results, as these were considered the closest species useful for comparisons. 

Colostrum IgG concentration has traditionally been considered the hallmark for evaluating colostrum quality before feeding to calf [3]. Concentration of IgG in maternal colostrum significantly affects the acquisition of passive immunity; thus, accurate measurement is essential to proper on-farm maternal colostrum management [27].

In the current study, the mean IgG in first milking maternal colostrum was slightly lower than that found in previous reports on Buffalo’s colostrum analysed by SDS-PAGE (71.4 mg/mL) [35] and higher than those reported for Egyptian buffaloes using sRID (33.2 mg/mL) [43] and Murrah buffaloes using indirect ELISA test (54.0 and 51.7 mg/mL) [44,45]. In the present study, the mean IgG colostrum concentration was 64.3 mg/mL that could be considered a good value since that 50 mg/mL is used as the standard for good quality colostrum in bovine cattle, as no standard for buffaloes is available. 

This cut-point has been widely used to define good and poor colostrum quality and reported in different original articles or reviews as an industry standard [3,41,46]. This standard value was based on the fact that for adequate PIT in dairy calves, a colostrum IgG mass of at least 150 to 200 g is needed to improve the chances of achieving success in passive transfer immunity (serum IgG ≥ 10 mg/mL obtained 1 to 7 d after birth) with 3 to 4 L of colostrum fed within 6 h after birth [47]. 

The average colostral IgG found in our study was similar to data recently reported in Holstein cows by Elshoaby et al. (2017) [48] 64.7 mg/mL, by Bartier et al. (2015) [40] 63.7 mg/mL, by Morrill et al. (2012) [26] 68.8 mg/mL, and by Løkke et al. (2016) [49] 59.7 mg/mL, but was lower than the IgG concentrations reported by Bielmann et al. (2010) [2] 94.4 mg/mL and Quigley et al. (2013) [27] 73.4 mg/mL. 

Several variables could affect the distribution of IgG also in the same species, such as animal management, nutritional practices that are known to affect colostrum quality, parity, the time of colostrum collection after calving, sample size, and use of non-analytical methods [3,10,36,50,51,52]. In our study, all the animals had good nutritional status and had their dry-off period respected, in order to prepare dams to the metabolic challenges inherent in the transition period [53,54].

The increase in the serum IgG found between T0 and T3 in the present study was consistent with previous findings in bovine calfs. Our findings were similar to those reported by Chaudary et al. (2018) (11.7 ± 0.75 mg/mL) [45] and Verma et al. (2018) (11.2 ± 0.7 mg/mL) [55] in buffalo calves, and lower than those reported by Mastellone et al. (2011) (31.0 ± 2.4 mg/mL) [56] in Mediterranean buffalo calves and in descendants of Murrah buffaloes by Souza et al. 2019 (35.3 ± 8.58 mg/mL) [5] and Souza et al. 2020 (34.5 ± 1.48 mg/mL) [35].

The differences found in IgG content could be attributable to the time of sampling and by the different analytical methods used to detect serum IgG concentrations that could affect the results. Radial immunodiffusion (sRID) is considered the gold standard for both colostrum and calf serum IgG evaluation [11]. However, sRID is a lab-based test that requires long processing times needing 18–24 h to obtain results, moreover it requires expensive reagents and it is susceptible to human error during the measurement of the ring diameter. Results can also vary between the different commercial kits used. Gelsinger et al. (2015) [25] found that ELISA and sRID are correlated, but ELISA values are consistently lower than sRID values. The findings reported in the present study in buffalo corroborate the suggestion that Brix refractometer could be a useful tool for the rapid assessing of colostrum quality and FPIT in ruminants. A close relationship between IgG content (determined by sRID or ELISA) and Brix measurements in colostrum and serum samples was found in bovine [2,28,37,38,40,49,57], goat, and sheep [29].

In the present study, we found colostrum %Brix ranged between 9.5 to 30.5 % with a mean value of 22.5 %Brix; our findings were similar to results reported by other authors [2,27,58] in dairy cattle. 

Published data on correlation coefficients between %Brix and IgG content in bovine colostrum vary broadly (*r* = 0.48–0.79) [1,27,40,57]. The high significant correlation index between %Brix and IgG content (*r_p_* = 0.75) in Buffalo colostrum found in the present study (Table 3; Figure 1 (b)) was slightly lower than that found in goats (*r* = 0.83, *p* < 0.001) and sheep (*r* = 0.75; *p* < 0.001) by Kessler et al. (2021) [29].

The positive correlations between Brix and IgG are not surprising given the respective relationships between %Brix and total protein content in colostrum (*r_p_* = 0.81; *p* < 0.001; Figure 1 (d)); this finding was similar to those detected in goats (*r_p_* = 0.89; *p* < 0.001) and sheep (*r* = 0.87; *p* < 0.001) [29]. Brix measurements are in fact related to total solids and thus indirectly to estimating IgG [40]. Consequently, correlations of %Brix with respective protein contents were even higher as in colostrum and serum samples. 

Serum TP represent a reliable indirect parameter used to evaluate PIT, because serum TP concentration has high sensitivity and specificity for the detection of FPIT [56]. The increase in total protein concentrations in calf serum is almost exclusively due to the absorption of Ig’s present in colostrum [59]. In this study, mean serum TP concentration in calves measured at 72 h reached values above the recommended threshold for an efficient PIT in dairy bovine calves (5.5 g/dL) and was consistent with serum TP concentrations reported in calves born from multiparous buffaloes [35]. The serum total protein concentration of samples from calves can be indicative of immunoglobulin concentrations, as ingestion of immunoglobulins will raise STP levels in the blood. As previously stated, mean serum total protein showed a high correlation with serum IgG (*r_p_* = 0.85, *p* < 0.001; Figure 2 (a)) and serum Brix (*r_p_* = 0.98, *p* < 0.001; Figure 2 (b)) and it could be utilized to evaluate FPIT [9,38]. Deelen et al. (2014) [38] suggested that the measurement of total proteins in the blood of dairy calves is an indirect measure of IgG concentration and it can be determined by refractometry.

In order to ensure that high quality colostrum is fed to calves, appropriate Brix score cut points need to be determined that strongly correspond to values over 50 mg of IgG/mL that are the acknowledged threshold for the classification of colostrum quality in cows. 

In the present study, 20 colostrum samples analysed by digital brix refractometer were over the standard bovine cut point of 50 mg/mL for Ig concentration (77%), while six colostrum samples had an IgG content < 50 mg/mL (23%), which was lower than that reported by Bartens et al. (2016; 34.7%) [50], Chigerwe et al. (2008; 32%) [36], Bartier et al. (2015; 29.1%) [40], and higher than the value reported by Bielmann et al. (2010; 7.7%) [2].

In previous reports, it has been suggested that %Brix values between 18 and 23% are appropriate cut-points for good quality colostrum [1,2,27,36]. 

In this study, the diagnostic test characteristics were established for Brix measurements on colostrum from first-calf heifers and calf serum samples. For colostrum samples taken from first-calf heifers, the highest combined values for sensitivity and specificity were seen at the 18% Brix cut point level (66.7 and 95.0%, respectively), indicating that the instrument had successfully identified colostrum samples with adequate Ig concentrations. Because it has been shown that colostrum of heifers is often lower for quality characteristics to that of older cows [60], it would seem reasonable to have a lower cut point level for first-calf heifers. Colostrum production has reported to be often lower in first lactation cattle, suggesting less mammary development and potentially reduced transport capacity for IgG into the mammary gland [60].

In previous studies on dairy cattle, at 50 mg of IgG/mL, the predicted Brix percentage (indicative of a break point for high-quality MC) ranged from 18% [26] and 21% [27], to 22% [2] or 23% [40]. Cows in their third or fourth parity or older usually have significantly higher levels of IgG per liter of colostrum than heifers or second parity cows [26,61], which is reflected in calves born to heifers having a greater risk for FPIT than calves born to multiparous cows [62]. From the current study, as well from the other studies cited, it is possible that parity of the dam has influenced the IgG concentration of colostrum. 

The value of 18% Brix resulted in the best combination of sensitivity and specificity and yielded the highest accuracy for our data. Brix score cut point level of 18% could be suggested as appropriate in first-lactation buffalo heifers for selection of good quality colostrum. Morrill et al., 2015, [1] reported that when analysing colostrum from Jersey cattle, a 18% Brix cut point may be used to determine colostrum quality. As suggested in previous reports, using a higher value may result in discarding quality colostrum. Bielmann et al. (2010) [2] in samples taken from first-calf heifers measured by the digital Brix refractometer, reported that the highest combined values for sensitivity and specificity were seen at the 21% Brix cut point level (97.2 and 75.0%, respectively). Dinsmore and Skidmore (2008) [63] found that an appropriate cut point level for first-lactation heifers was 23%, yielding a sensitivity of 84.6% and a specificity of 56.3%. 

In our study, sensitivity represents the aptitude to detect the proportion of poor-quality samples (<50 mg/mL) and Sp was defined as the proportion of good-quality samples (≥50 mg/mL) that were correctly identified as such. To compare the sensitivity and specificity of a device to a given cut point, it is important to verify what is meant by sensitivity and specificity in previous reports since some authors indicate the sensitivity as the ability to identify samples with concentrations ≥ 50 mg/mL, so it becomes inappropriate to compare results.

In the present study, the mean serum %Brix (9.6 ± 0.7) was slightly higher than the values reported by Morrill et al. (2013) [37] in Holstein calves. Deelen et al. (2014) [38] reported a mean serum %Brix concentration of 9.2% ± 0.9 with a range of 7.3 to 12.4%.

The evaluation of adequate passive transfer of immunity in the serum of calves has typically been conducted using a refractometer specifically calibrated for STP [38]. 

In the current study, as reported by Deleen et al. 2014 [38], we used the same refractometer to measure both serum %Brix and STP. Both measures were shown to have positive correlation to IgG values in our study, which suggests that the two methods are equivalent in efficacy in the samples tested. As such, it could be practical and beneficial for farmers to purchase a Brix refractometer, rather than a specific STP refractometer, to estimate IgG concentration of both maternal colostrum and calf serum [38]. This approach would allow farmers to monitor both colostrum quality and the success of PIT using one device [38]. 

In our study, 22 serum (85%) samples had IgG concentrations ≥ 10 mg/mL, consistent with adequate passive transfer. Four calves (15% of the subjects) had serum IgG concentration below the standard value suggested for bovines (<10 mg/mL), but they did not show signs of pathology during the trial. This data should be confirmed by a larger number of cases. Our findings are quite similar to the 10% reported by McCraken et al. (2017) [11], lesser than the 19.2% estimated by Beam et al. (2009) [64], and lower than the 37.1% reported by Trotz-Williams et al. (2008) [65] in a herd-level study. 

In the present study, the sensitivity and specificity of %Brix at 8.4% were 75.0 and 100.0%, respectively, and the accuracy was the highest calculated for the different cut points considered. So, this cut point could be considered as a reference value in order to discriminate between inadequate and adequate passive transfer of immunity in buffalo calves. McCraken et al. (2017) [11] suggested that cut-points of 7.1, 7.3, and 7.6% Brix resulted in similar percentages of samples being correctly classified as adequate passive transfer of immunity or failure of passive transfer of immunity and concluded that cut-points of 7.3 or 7.6% Brix are acceptable to evaluate adequate passive transfer in Jersey calves.

Previous data demonstrated that a digital refractometer is effective for use in rapid estimation of serum IgG concentrations in Holstein calves and a cut point value of 7.8% Brix was recommended to evaluate failure of passive transfer in calves without regard to breed differences [37].

Deelen et al. (2014) [38] suggested a useful value of 8.4% Brix to accurately evaluate FPIT in Holstein calves. These authors reported that sensitivity and specificity values of %Brix at 8.3% were 93.7 and 77.8%, respectively.

In conclusion, even though calves from first calving heifers seem to be more susceptible to FPIT, few samples in the current study had IgG levels < 10 mg/mL. For this reason, further evaluations of different populations are necessary. Probably, the careful monitoring of calves’ birth and the timely colostrum intake immediately after birth (on average within 3 hours) may have positively influenced the success of passive transfer. 

An adequate transfer of immunity in calves improves not only their health and growth in the first weeks of life, but also their further use as cows [66]. Furman-Fratczak et al. (2005) [62] reported that the main cause of FPIT or partial failure of passive transfer (PFPIT) (serum IgG = 5 to 10 mg/mL) in the calves seems to be poor vitality associated with dystocia and also low volume of ingested colostrum. The calves born to heifers would be more endangered than those born to multiparous cows.

Therefore, the amount of IgG that the calf consumes in the first hours after birth is one of the most important factors influencing successful transfer of passive immunity and calves’ health; therefore, it is essential for producers to have an on-farm tool available for the rapid assessment of colostrum quality.

## 5. Conclusions

An important objective of colostrum management is the possibility of a rapid on-farm quality evaluation. Data obtained in the present study suggested that a digital Brix refractometer could represent a useful tool for a timely estimation of IgG in Buffalo colostrum and also in calf’s serum, thus evaluating the success of the colostrum feeding program. Our data showed that colostrum %Brix were highly correlated with colostrum IgG content measured by ELISA. Moreover, colostrum %Brix showed a significant correlation with serum %Brix and other Brix measurements such as colostrum total protein and serum total protein.

Similarly, strong correlations were evidenced between Brix measurements on calf serum samples and IgG measured by ELISA. As previously found in dairy cows, the evaluation of serum total protein by Brix refractometry can be used to determine adequate passive transfer in calves.

For our data, the cut-point of 18% Brix showed the best accuracy in estimating colostrum quality from buffalo first-calf heifers. 

Moreover, a cut point of <8.4% for serum samples was more appropriate for detecting FPIT in buffalo calves. Digital Brix refractometry seems to be an affordable tool also in buffalo farms, allowing producers to use the same device for monitoring both colostrum quality and the success of passive transfer in calf serum.

However, further studies evaluating a higher number of animals would be needed in order to set specific cut point values.

## Figures and Tables

**Table 1:** Descriptive statistic of maternal colostrum (MC) from first-calf heifers (*n* = 26).

Item	Mean	SD	Min	Max
Colostrum Brix (%)	22.5	5.2	9.5	30.5
Colostrum ELISA- IgG (mg/mL)	64.9	29.3	13.0	110.0
Colostrum Total Protein (g/dL) *	14.3	3.0	10.0	20.5

* (Refractometry).

**Table 2:** Descriptive statistic of calves’ serum (*n* = 26) obtained at T3 (3 days after birth).

Item	Mean	SD	Min	Max
Serum Brix (%)	9.6	0.9	7.8	11.0
Serum ELISA-IgG (mg/mL)	11.1	2.0	6.0	14.5
Serum total protein (g/dL) *	6.3	0.7	4.8	7.5

* (Refractometry).

**Table 3:** Differences (Mean ± standard deviation) between calf serum Brix measurements (%, g/dL) and ELISA results (mg/mL) at T0 (at birth) and at T3 (72 hours after birth).

	T0	T3	*p*-Value
Serum Brix (%)	7.3 ± 0.5	9.6 ± 0.9	<0.001
Serum ELISA-IgG (mg/mL)	3.5 ± 0.4	11.1 ± 2.0	<0.001
Serum Total protein (g/dL) *	4.4 ± 0.5	6.3 ± 0.7	<0.001

* (Refractometry).

**Table 4:** Pearson correlation coefficients for selected variables.

Variable	Colostrum Brix (%)	Serum Brix (%)	Colostrum Total Protein (g/dL)	Serum Total Protein (g/dL)	Colostrum ELISA-IgG (mg/mL)	Serum ELISA-IgG (mg/mL)
Colostrum Brix (%)	1	0.82 ***	0.81 ***	0.79 ***	0.75 ***	0.74 **
Serum Brix (%)		1	0.78 ***	0.98 ***	0.62 *	0.85 ***
Colostrum total protein (g/dL)			1	0.75 ***	0.65 *	0.71 **
Serum total protein (g/dL)				1	0.60 *	0.85 ***
Colostrum ELISA-IgG (mg/mL)					1	0.75 **
Serum ELISA-IgG (mg/mL)						1

* *p*-value ≤ 0.05; ** *p*- value ≤ 0.01; *** *p*-value ≤ 0.001.

**Table 5:** Diagnostic test characteristics for colostrum samples from first calf heifers. Sensitivity (Se), specificity (Sp), positive predictive value (PPV), negative predictive value (NPV), accuracy, false positive (FP), and false negative (FN) for different Brix refractometer cut-points compared with 50 mg/mL of IgG as measured by ELISA.

Cut Point Value	Se (%)	Sp (%)	Se + Sp ^1^	PPV ^2^ (%)	NPV ^3^ (%)	Accuracy ^4^ (%)	^5^ FP	^6^ FN
18%	66.7 (45.6–83.0)	95.0 (76.9–99.5)	1.62	80.0 (59.2–92.2)	90.5 (71.1–97.9)	88.0	1	2
19%	75.0 (53.9–89.0)	83.3 (62.8–55.9)	1.58	57.1 (36.7–75.6)	89.5 (69.9–97.5)	80.8	3	2
20%	83.3 (62.8–94.2)	75.0 (53.9–89.0)	1.58	50.0 (30.4–69.6)	93.8 (75.2–99.2)	76.9	5	1
23%	93.3 (74.7–99.0)	45.5 (26.0–65.6)	1.38	70.0 (48.9–85.5)	83.3 (62.8–94.2)	73.0	6	1

Se = sensitivity; ^1^ Sp = specificity; ^2^ PPV = positive predictive value; ^3^ NPV = negative predictive value. Sensitivity and specificity percentages converted into a unit value. 95% confidence interval shown in parentheses for Se, Sp, PPV, and NPV. ^4^ Accuracy = percentage of colostrum samples correctly classified as good (≥50 mg/mL) or poor (<50 mg/mL) quality. ^5^ FP = false positive; ^6^ FN = false negative.

**Table 6:** Diagnostic test characteristics for serum samples from calves. Sensitivity (Se), specificity (Sp), positive predictive value (PPV), negative predictive value (NPV), accuracy, false positive (FP), and false negative (FN) for different Brix refractometer cut-points compared with 10 mg/mL of IgG as measured by ELISA.

Cut Point Value	Se (%)	Sp (%)	Se + Sp ^1^	PPV ^2^ (%)	NPV ^3^ (%)	Accuracy ^4^ (%)	FP ^5^	FN ^6^
7.8%	25.0 (11.0-46.1)	100.0 (84.0-100.0)	1.25	100.0 (84.0-100.0)	88.0 (68.2-96.7)	88.5	0	3
8.0%	33.3 (17.0-54.4)	100.0 (84.0-100.0)	1.33	100.0 (84.0-100.0)	92.0 (73.0-98.5)	92.3	0	2
8.4%	75.0 (53.9-89.0)	100.0 (84.0-100.0)	1.75	100.0 (84.0-100.0)	95.7 (77.7-99.7)	96.1	0	1

Se = sensitivity; Sp = specificity; ^1^ Sensitivity and specificity percentages converted into a unit value; ^2^ PPV = positive predictive value; ^3^ NPV = negative predictive value. 95% confidence interval shown in parentheses for Se, Sp, PPV, and NPV. ^4^ Accuracy = percentage of samples correctly classified as high (≥10 mg of IgG/mL) or low (<10 mg of IgG/mL). ^5^ FP = false positive; ^6^ FN = false negative.

## Data Availability

The data presented in this study are available on request from the corresponding author.
